# Measuring population health: costs of alternative survey approaches in the Nouna Health and Demographic Surveillance System in rural Burkina Faso

**DOI:** 10.3402/gha.v8.28330

**Published:** 2015-08-07

**Authors:** Henrike Lietz, Moustapha Lingani, Ali Sié, Rainer Sauerborn, Aurelia Souares, Yesim Tozan

**Affiliations:** 1 Institute of Public Health, Heidelberg University, Heidelberg, Germany; 2 Centre de Recherche en Santé de Nouna, Nouna, Burkina Faso; 3 Steinhardt School of Culture, Education and Human Development and College of Global Public Health, New York University, New York, NY, USA

**Keywords:** cost analysis, Health and Demographic Surveillance Systems, health surveys, data collection, survey approaches, Burkina Faso

## Abstract

**Background:**

There are more than 40 Health and Demographic Surveillance System (HDSS) sites in 19 different countries. The running costs of HDSS sites are high. The financing of HDSS activities is of major importance, and adding external health surveys to the HDSS is challenging. To investigate the ways of improving data quality and collection efficiency in the Nouna HDSS in Burkina Faso, the stand-alone data collection activities of the HDSS and the Household Morbidity Survey (HMS) were integrated, and the paper-based questionnaires were consolidated into a single tablet-based questionnaire, the Comprehensive Disease Assessment (CDA).

**Objective:**

The aims of this study are to estimate and compare the implementation costs of the two different survey approaches for measuring population health.

**Design:**

All financial costs of stand-alone (HDSS and HMS) and integrated (CDA) surveys were estimated from the perspective of the implementing agency. Fixed and variable costs of survey implementation and key cost drivers were identified. The costs per household visit were calculated for both survey approaches.

**Results:**

While fixed costs of survey implementation were similar for the two survey approaches, there were considerable variations in variable costs, resulting in an estimated annual cost saving of about US$45,000 under the integrated survey approach. This was primarily because the costs of data management for the tablet-based CDA survey were considerably lower than for the paper-based stand-alone surveys. The cost per household visit from the integrated survey approach was US$21 compared with US$25 from the stand-alone surveys for collecting the same amount of information from 10,000 HDSS households.

**Conclusions:**

The CDA tablet-based survey method appears to be feasible and efficient for collecting health and demographic data in the Nouna HDSS in rural Burkina Faso. The possibility of using the tablet-based data collection platform to improve the quality of population health data requires further exploration.

Understanding the changes in population health patterns and trends is important for the planning, monitoring, and evaluation of health programs and policies. Timely and accurate health data produced by well-functioning health information systems are essential for evidence-based policy-making and resource allocation at national and sub-national levels (1, 2). Health information systems in low-and middle-income countries (LMICs) are often weak. The representativeness of routine health facility data in LMICs is severely limited because of low coverage and utilization of health care (3, 4). Other data sources for population health include vital registration, national censuses, household surveys, national health accounts, and health research (5–7). In such settings, Health and Demographic Surveillance Systems (HDSS), when available, and household health surveys have become a common method for collecting data on population and health (8). These surveys provide complementary data to health-facility-based reporting systems or supplementary data to large-scale surveys conducted at usually long intervals, such as Demographic and Health Surveys (9).

As a longitudinal data collection process, the HDSS monitors the population dynamics and the health status of a population in a geographically well-defined area. The HDSS provides a platform for population-based health research and evaluation of health tools and interventions relevant to local health priorities and needs (10). Preceded by a baseline household census, vital events (e.g. births, marriages, migration, and deaths) are tracked and updated regularly (Vital Event Registration, VER), usually three to four times a year (11). However, HDSS sites are not representative of the entire region or the country. Several HDSS sites now implement the verbal autopsy method to collect data on causes of death (12). Most deaths in LMICs occur outside the health care system, and vital registration systems are often non-existent or weak. This tool is, therefore, of particular importance in collecting mortality data. HDSS sites also provide a platform to other longitudinal data collection efforts, such as the World Health Organization (WHO) Study on Global AGEing and Adult Health (SAGE) and surveillance systems for chronic non-communicable disease risk factors (10, 13). Many HDSS sites also undertake morbidity research studies (14–16). There are currently more than 40 HDSS sites in 19 different countries, organized by the International Network for the Demographic Evaluation of Populations and Their Health (INDEPTH) (17). The question of how to integrate data collection activities is important for all HDSS sites across the world. Given the high running costs of HDSS sites, the financing of HDSS activities is also of major importance, and adding external health surveys to the system, in general, is challenging for HDSS sites.

Household surveys are a common tool for collecting population-based data. These surveys can be cross-sectional, where a sample of households is surveyed on a single occasion, or longitudinal, where the same group of households (panel) is examined on two or more occasions (waves). They may include only interviews (health interview surveys) or incorporate biological and physiological biomarkers (health examination surveys) (18). The recall period in most surveys is 1 month. Recall bias and factors such as age, sex, education, and socio-cultural characteristics affect the reliability of self-reported health data (19, 20). For example, given the same level of objective health, less educated people are more likely to report more episodes of illness than more educated people (20, 21). By definition, cross-sectional surveys are not conducted on a recurrent basis because of the large sample size requirements. Panel surveys are particularly expensive due to the costs associated with panel maintenance (22).

Nevertheless, all of these instruments have limitations. The issue then becomes how to comprehensively and effectively assess all of the health dimensions of a population, including disease and illness, along with an understanding of changing population characteristics in LMICs. HDSS surveillance rounds and a Household Morbidity Survey (HMS) were conducted independently in the Nouna HDSS until 2011. (No HMS took place between 2012 and 2014.) To improve data quality and collection efficiency (23), these stand-alone data collection activities were integrated and the paper-based questionnaires were consolidated into a single tablet-based questionnaire, namely the Comprehensive Disease Assessment (CDA). This new survey will be implemented in 2016 after a pre-test phase, which included the training of all involved fieldworkers and supervisors, the development and testing of the software, and a field pre-test of the integrated survey approach. The pre-test phase provided information about the challenges and costs associated with implementing a tablet-based survey in this HDSS site. This study involves a comparative analysis of survey costs. The aims are to estimate and compare the implementation costs of the two different survey approaches, which are stand-alone HDSS and HMS versus integrated CDA, for measuring population health in the Nouna HDSS in rural Burkina Faso.

## Methods

### Study area

**Fig. 1:**
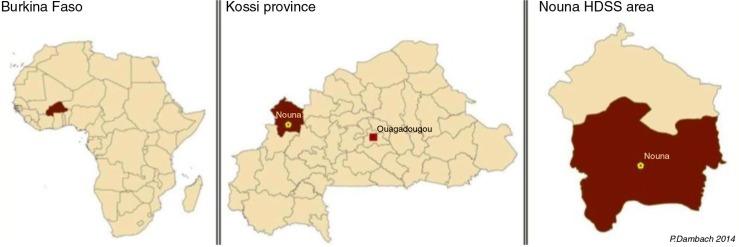
Location of the Nouna HDSS in north-western Burkina Faso.

The Nouna Health District is located in the Kossi Province, in the north-western part of Burkina Faso, about 300 km from the capital Ouagadougou (Fig. 1) (3, 24). A total of 89,000 inhabitants with diverse ethnic and religious backgrounds live in this area, and 30% of the population resides in the semi-urban town of Nouna. The region is a dry orchard savannah, and the predominantly rural population depends on subsistence farming and cattle raising for their livelihood (24).

The Nouna HDSS has been a member of the INDEPTH network since 1992. Every year a total of 10,000 households (about 60,000 people) are interviewed three times to collect data on vital events using paper-based questionnaires. Moreover, verbal autopsy is conducted on those households. A permanent team of 14 interviewers and four supervisors undertake HDSS data collection activities routinely. Since 2002, a panel of 1,000 HDSS households have been surveyed once a year to collect data on the health and socio-economic status of all household members, using paper-based questionnaires (24). An additional 24 interviewers and 12 supervisors were hired for a period of 2 months to administer the HMS. Both HDSS surveillance rounds and the HMS were independently implemented until recently, requiring manpower, time, training, and other resources.

### Comprehensive Disease Assessment

The main objective for developing the CDA survey was to improve data quality and collection efficiency in the Nouna HDSS by integrating stand-alone data collection activities and consolidating paper-based questionnaires into a single questionnaire. This also provided an opportunity to move from traditional paper-based interviewing to tablet-based interviewing, which has been gaining popularity in epidemiological and public health research. Comprehensive validity and readability checks were built into the newly developed tablet-based questionnaire to ensure data quality. Twenty fieldworkers and five supervisors were trained to use tablet computers and new survey software. Moreover, six additional interviewers were hired to enlarge the team because of the increased workload (morbidity module integrated with the HDSS data collection). The tablet-based survey instrument was pre-tested to eliminate software bugs, to make it as accessible as possible for interviewers and to increase its acceptance rate within the population. The CDA targets the panel of 1,000 HDSS households that participated in the 2010 HMS (unpublished observation). The integrated approach allows for interviewing participating households for health status when a VER interview is conducted during a routine HDSS round. The panel of households is randomly spread throughout the year to capture seasonal variations of health. In essence, the data collection activity for the HMS is now nested within the routine HDSS rounds for this panel of households. The CDA survey is envisioned to be a permanent data collection tool in the Nouna HDSS. Its main advantage is that HDSS households are to be visited a maximum of three times a year, potentially reducing the time burden of data collection to both interviewers and respondents.

### Cost analysis

Financial costs of the stand-alone (HDSS and HMS) and integrated (CDA) survey approaches were estimated from the perspective of the implementing agency (25), namely the Centre de Recherche en Santé de Nouna (CRSN). Financial costs constitute all of the cash expenditures incurred by the CRSN for the implementation of the stand-alone and integrated surveys in the Nouna HDSS. Cost data for HDSS and HMS were collated from the 2010 financial expenditure records of the CRSN in West African Francs (CFA) and inflated to 2014 CFA using the consumer price index for Burkina Faso (26). Cost data for CDA were estimated based on its actual pre-testing expenditures as presented in the 2014 financial expenditure records of the CRSN in local currency and the planning and budgeting exercise for survey implementation in 2014. All costs in local currency were converted to US Dollars (US$) using the 2014 average exchange rate (1 US$=526 CFA) (27). The accountant of the CRSN validated all of the cost data collated from annual financial expenditure records.

Survey implementation costs are commonly divided into two categories: fixed and variable costs (28). Fixed costs are survey costs that do not change with the size of the surveyed population, whereas variable costs depend upon it. Following the structure of each survey, we identified the fixed and variable costs associated with the implementation of the stand-alone and integrated surveys in the Nouna HDSS.

The fixed costs included time costs of CRSN lead staff, who oversaw survey implementation, and rental and housekeeping costs of CRSN office space, including internet costs. Because CRSN lead staff were involved with multiple projects simultaneously, the percentage of time devoted to each survey implementation was used as a basis to allocate staff time costs (25). For example, a staff member working 15 days per month on a project would have spent 180 out of a total of 240 work-days a year on it. His or her time cost on the project was calculated by multiplying staff member's annual salary by 0.75 (180/240). The same calculation was applied in allocating rental costs of office space and housekeeping costs for each survey implementation.

The variable costs included time costs of interviewers undertaking data collection, supervisors’ monitoring of data collection activities, CRSN staff undertaking data management activities (e.g. data entry, validation, cleaning, and basic descriptive analysis and data storage), rental and housekeeping costs of office space used for data management activities, and costs of survey consumables (e.g. stationery and other supplies). These variable costs were estimated using an ingredients approach by identifying the number of units employed in implementing each survey and multiplying these by their unit prices. Costs of transportation included fuel and overnight stays of field personnel (i.e. interviewers and supervisors). Field personnel received a monthly allowance for transportation and use of personal motorbikes and vehicles. Such transportation costs also applied to CDA. In contrast to HDSS and CDA, HMS required hiring and training of new field workers because it was implemented only once a year and was not regarded as a standardized data collection method.

Lastly, we estimated the set-up costs of the CDA survey. These included the costs of developing and pre-testing the survey instrument and procurement costs of tablets and batteries. Training costs were part of the survey set-up costs because the CDA was intended to be a standardized data collection tool, similar to the HDSS. In summary, we estimated two categories of costs, namely survey set-up costs and survey implementation costs, to arrive at the total costs associated with each of the two survey approaches in the Nouna HDSS.

## Results

Table 1 summarizes the estimated implementation costs of the stand-alone surveys and the integrated survey by cost category in the Nouna HDSS in Burkina Faso. For the HDSS survey, the implementation cost amounted to US$186,317, the majority of which was variable costs (US$175,750; 94%). The key cost drivers of the variable costs were field personnel (US$81,176; 44%) and data management activities (US$55,573; 30%), followed by transportation (US$32,780; 18%). For HMS, the implementation costs amounted to US$65,324. As for the HDSS, the majority of the implementation costs were variable (US$62,839; 96%), and the key cost driver was field personnel (US$28,493; 47%). Data management (US$10,958; 17%), transportation (US$10,942; 17%), and training of field personnel (US$10,036; 15%) costs contributed approximately equally to the implementation cost of HMS. The traditional paper-based format of the stand-alone surveys required intensive efforts for data entry and validation, particularly for HDSS where 10,000 households were surveyed in any given year. The combined survey implementation costs of the two stand-alone surveys amounted to US$251,641 in 2010.

**Table 1:** Annual costs of survey implementation for the stand-alone and the integrated surveys in the Nouna HDSS, Burkina Faso^a^

	Stand-alone surveys			Integrated survey
		
Cost category	HDSS^b^ US$ (%)	HMS^c^ US$ (%)	HDSS+HMS US$ (%)	CDA^d^ US$ (%)
Fixed costs	10,567 (5.67)	2,431 (3.72)	12,998 (5.17)	12,393 (5.99)
CRSN personnel (lead team)	8,735 (4.69)	1,767 (2.70)	10,502 (4.17)	10,354 (5.00)
CRSN office space	436 (0.23)	82 (0.13)	518 (0.21)	485 (0.23)
CRSN housekeeping	1,396 (0.75)	582 (0.89)	1,978 (0.79)	1,554 (0.75)
Variable costs	175,750 (94.33)	62,839 (96.28)	238,643 (94,83)	194,544 (94.01)
Field personnel	81,176 (43.57)	28,493 (46.62)	109,669 (43.58)	128,555 (62.12)
Interviewers	56,441 (13.28)	16,126 (24.69)	72,567 (28.84)	92,413 (17.47)
Supervisors	24,735 (30.29)	12,367 (18.93)	37,102 (14.74)	36,142 (44.66)
Consumables	6,221 (3.34)	2,464 (3.77)	8,683 (3.45)	10,726 (5.18)
Bags	144 (0.08)	246 (0.38)	390 (0.15)	285 (0.14)
Pens	2 (0.00)	3 (0.00)	5 (0.00)	4 (0.00)
Torches	36 (0.02)	62 (0.09)	98 (0.04)	76 (0.04)
Phone cards	5,740 (3.08)	1,640 (5.51)	7,380 (2.93)	9,126 (4.41)
Battery for torches	24 (0.01)	41 (0.06)	65 (0.03)	38 (0.02)
Rain jackets	191 (0.10)	328 (0.50)	519 (0.21)	304 (0.15)
Folders	84 (0.05)	144 (0.22)	228 (0.09)	133 (0.06)
Covers for tablets	–	–	–	760 (0.37)
Transportation	32,780 (17.59)	10,942 (16.75)	43,722 (17.37)	50,646 (24.47)
Monthly allowance for interviewers	25,400 (13.63)	7,252 (11.10)	32,652 (12.98)	40,380 (19.51)
Monthly allowance for supervisors	7,380 (3.96)	3,690 (5.65)	11,070 (4.40)	10,266 (4.96)
Data management	55,573 (29.83)	10,958 (16.77)	66,531 (26.44)	4,617 (2.23)
Questionnaire printing	11,217 (6.02)	349 (0.53)	11,566 (4.60)	–
Data clerks	19,782 (10.62)	2,473 (3.79)	22,255 (8.84)	–
Data clerk supervisors	6,789 (3.63)	847 (1.30)	7,615 (3.03)	–
Data quality manager	7,891 (4.21)	2,630 (4.03)	10,521 (4.18)	2,195 (1.06)
IT manager	8,634 (4.63)	4,317 (6.61)	12,951 (5.15)	1,709 (0.83)
IT office space	1,281 (0.69)	342 (0.52)	1,623 (0.64)	713 (0.34)
Training^e^	–	10,036 (15.36)	10,036 (3.99)	–
Trainers	–	385 (0.59)	385 (0.15)	–
Interviewers	–	3,091 (4.73)	3,091 (1.23)	–
Supervisors	–	4,032 (6.17)	4,032 (1.60)	–
Catering	–	2,528 (3.87)	2,528 (1.00)	–
Total	186,317	65,324	251,641	206,937

HDSS=Health and Demographic Surveillance Systems; HMS=Household Morbidity Survey; CDA=Comprehensive Disease Assessment; CRSN=Centre de Recherche en Santé de Nouna.

a All costs are in 2014 US$.

b HDSS: permanent data collection tool (implementation over 12 months).

c HMS: non-permanent data collection tool (implementation over 2 months).

d CDA: permanent data collection tool (implementation over 12 months).

e Training: HMS requires a 2-week training period every year.

The implementation cost of the CDA survey was estimated at US$206,937. Similar to HDSS and HMS, the variable costs constituted the majority of the survey implementation costs, accounting for 94% of it (US$194,544). The key cost drivers were field personnel (US$128,555; 62%), followed by transportation (US$50,646; 25%). The use of newly developed software and tablets considerably reduced the costs of data management activities, which only constituted 2% (US$4,617) of the survey implementation cost. The CRSN incurred set-up costs, which amounted to US$68,196 (Table 2). The procurement of tablets and batteries (US$50,349; 66%) was the key cost driver, followed by the development of new software (US$11,002; 14%). The CRSN information technology (IT) manager worked for about 3 months, and an IT consultant worked for an additional month, on software programing. Because tablet-based interviewing was a newly introduced data collection method, a pre-test was conducted. The pre-test costs amounted to US$8,365 (11%). The CRSN spent an additional US$8,929 (11%) on training of the field staff on the use of the new survey software and tablets.

**Table 2:** Survey set-up costs of the integrated survey (CDA) in the Nouna HDSS, Burkina Faso^a^

Cost category	Quantity	Unit cost	Unit	Attributed use	Total (%)
Software development					11,002 (14.37)
IT specialist	1	1,709	Per month	3 months	5,126 (6.69)
IT consultant	1	2,852	Per month	1 month	2,852 (3.72)
Travel to Ouagadougou	7	432	Round trip	1	3,024 (3.95)
Procurement					50,349 (65.76)
Tablets	21	1,885	Tablet	1	39,585 (51.70)
Batteries	36	299	Battery	1	10,764 (14.06)
Pre-test^b^					8,365 (10.64)
Trainer salary	1	1,709	Participant	0.5 month	1,701 (2.22)
Supervisors salary	4	602	Participant	1 month	2,408 (3.14)
Interviewers salary	6	385	Participant	1 month	2,310 (2.94)
Catering	11	10	Participant	1	1,100 (1.40)
Monthly transportation allowance for supervisors	4	171	Participant	0.5 month	342 (0.43)
Monthly transportation allowance for interviewers	6	168	Participant	0.5 month	504 (0.64)
Training^c^					8,929 (11.35)
Trainer salary	1	1,709	Participant	0.5 month	855 (1.09)
Supervisor salary	5	602	Participant	0.5 month	1,506 (1.91)
Interviewer salary	20	385	Participant	0.5 month	3,851 (4.90)
Catering	26	9.50	Participant	10 days	2,470 (3.14)
Training materials	26	9.50	Participant	1	247 (0.31)
Total					68,196

IT=information technology.

a All costs are in 2014 US$.

b Pre-test duration: 1 month.

c Training duration: 2 weeks.

The two survey approaches, 1) stand-alone, paper-based HDSS and HMS vs. 2) integrated, tablet-based CDA, varied considerably in terms of the numbers of personnel involved (Table 3) and their overall survey implementation costs (Table 1). The implementation cost of the CDA survey ($206,937) was estimated to be less than that of the combined two stand-alone surveys ($251,641), this being a difference of US$44,704. While the fixed costs of survey implementation were similar across the two survey approaches, there were considerable variations in the variable costs. Although the annual cost of field personnel for the CDA survey was estimated to be higher than that of the stand-alone surveys (CDA: US$128,555 vs. HDSS+HMS: US$109,669), variable costs were less overall (CDA: US$194,544 vs. HDSS+HMS: US$243,643). High field personnel costs for the CDA survey could be explained by the increased number of permanent field workers hired over a 12-month period. Consequently, costs of survey consumables and transportation were also higher for the CDA survey compared to the stand-alone surveys (Table 1), even if the overall numbers of visits were reduced for the households that were also sampled for the HMS. Costs of data management for CDA were considerably lower than for the stand-alone surveys. This was mainly because the tablet-based survey format eliminated the need for costs associated with data entry and cleaning. Because the CDA survey would be implemented by permanent field staff as a standardized data collection tool, it would not require annual routine training for all field personnel. A team of 29 people, including field and data management personnel, would be established and trained to conduct the CDA survey under the supervision of the CRSN lead staff. The CDA survey would be implemented using fewer field personnel than required for the stand-alone surveys. Therefore, the CDA survey could potentially allow for capacity building activities at the CRSN and for more efficient use of staff time.

**Table 3:** Personnel needs and unit costs for the stand-alone and integrated surveys in the Nouna HDSS, Burkina Faso^a^

	HDSS		HMS		CDA	
			
Category of personnel	Quantity	Unit cost	Quantity	Unit cost	Quantity	Unit cost
Field personnel						
Interviewers	14	336	24	336	20	385
Supervisors	4	515	12	515	5	602
Data management						
Data entry clerks	6	275	3	275	–	–
Data entry supervisors	2	282	1	282	–	–
Data quality manager	1	658	2	658	3	732
IT manager	1	1,739	1	1,739	1	1,709

HDSS=Health and Demographic Surveillance Systems; HMS=Household Morbidity Survey; CDA=Comprehensive Disease Assessment; IT=information technology.

a All costs are in 2014 US$.

Our cost analysis showed that the CRSN spent about US$25 per household visit on data collection using the stand-alone surveys. The CDA survey was estimated to reduce the cost per household visit to about US$21 for collecting an equivalent amount of information from 10,000 households in the Nouna HDSS (Table 4).

**Table 4:** Annual cost per household visit for the stand-alone and integrated surveys in the Nouna HDSS, Burkina Faso^a^

	Stand-alone surveys		Integrated survey	
		
Category	HDSS	HMS	HDSS+HMS	CDA
Annual total cost	186,317	65,324	251,641	206,937
Number of interviewed household	10,000	1,000	10,000	10,000
Annual cost per household visit	19	65	25	21

HDSS=Health and Demographic Surveillance Systems; HMS=Household Morbidity Survey; CDA=Comprehensive Disease Assessment; IT=information technology.

a All costs are in 2014 US$.

## Discussion

This is the first comparative cost analysis of the two survey approaches used to measure population health in the Nouna HDSS in rural Burkina Faso. Most HDSS sites incur high running costs due to routine surveillance activities in any given year. The implementation of cross-sectional or panel surveys in HDSS sites for health research studies requires additional costs and manpower. Our results suggested that the integrated survey approach in this HDSS site, using the CDA survey, would lead to an estimated annual cost saving of about US$45,000 compared to the stand-alone survey approach where the HDSS and HMS were run independently. It is estimated that the tablet-based CDA survey could substantially reduce the high costs of data entry and management activities compared with stand-alone paper-based surveys. Our findings were in line with the findings of other studies that assessed the costs associated with developing and implementing surveys using tablet computers and other so-called smart devices (29–34). We intended our analysis to provide a basis for considering the personnel time and expertise required and the set-up costs that may be involved with the development of a tablet-based survey in other HDSS sites. However the estimated cost-savings shown here could potentially offset some of these set-up costs. Also new modules could easily be added to the CDA survey at marginal additional costs.

In addition to these estimated cost-savings, the tablet-based CDA survey has the potential to improve the quality of health data collected in the Nouna HDSS. Advanced mobile devices, such as smartphones and tablet computers, have been shown to overcome important limitations of paper-based survey tools (35). The use of such devices can make field-based data more readily available for analysis and review (30), improve data accuracy by reducing data collection and entry errors (33), and help prevent data loss problems (32, 33). A permanent team of interviewers who collect health data could improve rapport with respondents and potentially decrease recall and reporting bias in self-reported answers (20, 36). Lastly, the use of tablet computers might increase motivation among interviewers and the acceptance rate among respondents. There is evidence of this from a number of studies showing that tablet-based interviewing is likely to reduce the time burden of data collection for both groups (37–40). The organization of data collection in the field using permanent interviewers and supervisors can help improve the data quality.

Given the longitudinal design of the CDA survey, health status data will be collected at multiple time points during the course of a year, capturing seasonal variations in disease patterns. The data from the CDA survey will be used to generate reliable estimates of the local disease burden (36).

A limitation of our cost analysis was that we estimated the financial commitment required to establish and maintain the CDA survey based on the pre-test costs and the budgeting exercise in 2014. We compared its implementation cost to those of stand-alone surveys, based on the 2010 financial expenditure records of the CRSN. In this costing analysis, therefore, we made conservative assumptions. For instance, we assumed that the monthly allowance given to interviewers and supervisors for transportation would remain the same, although HDSS+HMS households would be visited less frequently for the CDA survey, and an optimized time schedule might further reduce these costs. The feedback from supervisors following the pre-test supported this assumption. It is also likely that the actual number of field personnel to conduct the CDA might decrease or increase, based on implementation experience. However, the CDA is expected to require much less field personnel than the stand-alone approaches combined. Lastly, there is no long-term experience with tablet-based survey activities. There could potentially be additional costs to maintain the tablet-based data collection platform (e.g. software updates and tablet repairs) and to provide field personnel with technical support and continuous training. Furthermore, the internet connectivity and power cut-offs will challenge the tablet-based data collection and storage. This will require good field organization to allow the interviewers to charge the tablets every day. Car batteries will be provided, and interviewers will have a second battery at their disposal to be able to collect data 10 hours each day. Screens also have a special transparent slide to allow interviewers’ better vision in the sun, as the interviews will be conducted outside.

To date there have been no published studies which have evaluated the costs of survey integration in HDSS sites. The few available studies, where health modules were added to HDSS surveillance rounds, focused on the quality of the collected health data, but not on the technical or economic aspects of survey integration (10, 13).

In areas where vital events registration and health information systems are weak or non-existent, the HDSS provides a well-structured platform to collect valid and reliable population-based data. The longitudinal CDA survey is one option to provide a more comprehensive measurement of health, including mortality, morbidity, and self-reported health measurement, and to reduce the overall costs of survey implementation in the Nouna HDSS, as suggested by our analysis. These findings could also apply to other HDSS sites. Nevertheless, further research is needed to assess the validity of the data collected using tablet computers, in terms of data accuracy and user preferences, including both interviewers and respondents (30). Furthermore, the costs of the CDA survey should be re-evaluated following its implementation as a routine survey tool as its costs were estimated based on the pre-test phase.

## Conclusion

There is a lack of valid and reliable information on population health in LMICs. This costing analysis estimated that the CDA survey would reduce the annual costs of survey implementation by about US$45,000. The tablet-based data collection platform is likely to increase the quality of population and health data collected. The CDA survey appears to be a feasible and efficient method of data collection in the Nouna HDSS in rural Burkina Faso that deserves further exploration.
